# MicroRNA, Pm-miR-2305, Participates in Nacre Formation by Targeting Pearlin in Pearl Oyster *Pinctada martensii*

**DOI:** 10.3390/ijms160921442

**Published:** 2015-09-07

**Authors:** Yu Jiao, Zhe Zheng, Rongrong Tian, Xiaodong Du, Qingheng Wang, Ronglian Huang

**Affiliations:** 1 Fishery College, Guangdong Ocean University, Zhanjiang 524025, Guangdong, China; E-Mails: jiaoyu0309@163.com (Y.J.); haidazhengzhe@163.com (Z.Z.); Rongrong_Tian619@126.com (R.T.); wangqingheng@163.com (Q.W.); hrl8849@163.com (R.H.); 2 Guangdong Technology Research Center for Pearl Aquaculutre and Process, Guangdong Ocean University, Zhanjiang 524025, China

**Keywords:** *Pinctada martensii*, biomineralization, nacre formation, miR-2305

## Abstract

MicroRNAs (miRNAs) are noncoding RNA molecules that function as negative regulators of target genes. In our previous research, 258 pm-miRNAs were identified in *Pinctada martensii* by Solexa deep sequencing. Pm-miR-2305 was one of the identified pm-miRNAs with a potential function in biomineralization. In the present study, the precursor of pm-miR-2305 was predicted with 96 bp, containing a characteristic hairpin structure. Stem-loop qRT-PCR analysis indicated that pm-miR-2305 was constitutively expressed in all the tissues (adductor muscle, gill, mantle, hepatopancreas, foot, and gonad) of *P. martensii* and was highly expressed in the foot. After the over-expression of pm-miR-2305 in the mantle by mimics injection into the muscle of *P. martensii*, nacre demonstrated disorderly growth, as detected by scanning electron microscopy. Dual luciferase reporter gene assay indicated that pm-miR-2305 mimics could significantly inhibit the luciferase activity of the reporter containing the 3′UTR of the pearlin gene. Western blot analysis demonstrated that the protein expression of pearlin was down-regulated in the mantle tissue after the over-expression of pm-miR-2305. Therefore, our data showed that pm-miR-2305 participated in nacre formation by targeting pearlin in *P. martensii*.

## 1. Introduction

MicroRNAs (miRNAs) are short (approximately 21-nucleotide) noncoding RNAs that are direct negative regulators of gene expression by binding to specific sequences within a target mRNA [1]. MiRNAs repress cellular protein levels to provide a sophisticated parameter of gene regulation that coordinates a broad spectrum of biological processes [2,3]. Inhibition of mRNA translation by miRNAs has proven to be an important regulator of biomineralization, such as bone resorption activity and bone homeostasis, in the adult skeleton [2,3]. MiRNAs control multiple layers of gene regulation for bone development, from the initial response of progenitor cells to the structure and metabolic activity of the mature tissue [2,3].

Nacre formation, similar to bone formation, is also a typical biomineralization process [4]. To investigate the mechanism that controls the biomineralization process in nacre formation, various matrix proteins have been extracted and functionally studied [1,5,6,7]. However, nacre formation is a very complex, precise process, and the expression of each related protein is subject to fine regulation [8]. Consequently, regulators involved in nacre formation should also be given attention. Identification and characterization of the related miRNAs can contribute to the understanding of the mechanism underlying nacre formation.

*Pinctada martensii* is the main species cultured for marine pearl production in China and Japan. In our previous research, using Solexa deep sequencing technology, we obtained 258 pm-miRNAs and identified their potential functions in biomineralization [8]. Pm-miR-2305 was one of the identified pm-miRNAs with potential function in biomineralization [8]. The aim of this report was to verify the function and mechanism of pm-miR-2305 in nacre formation.

## 2. Results

### 2.1. Precursor Prediction of Pm-miR-2305

**Figure 1:**
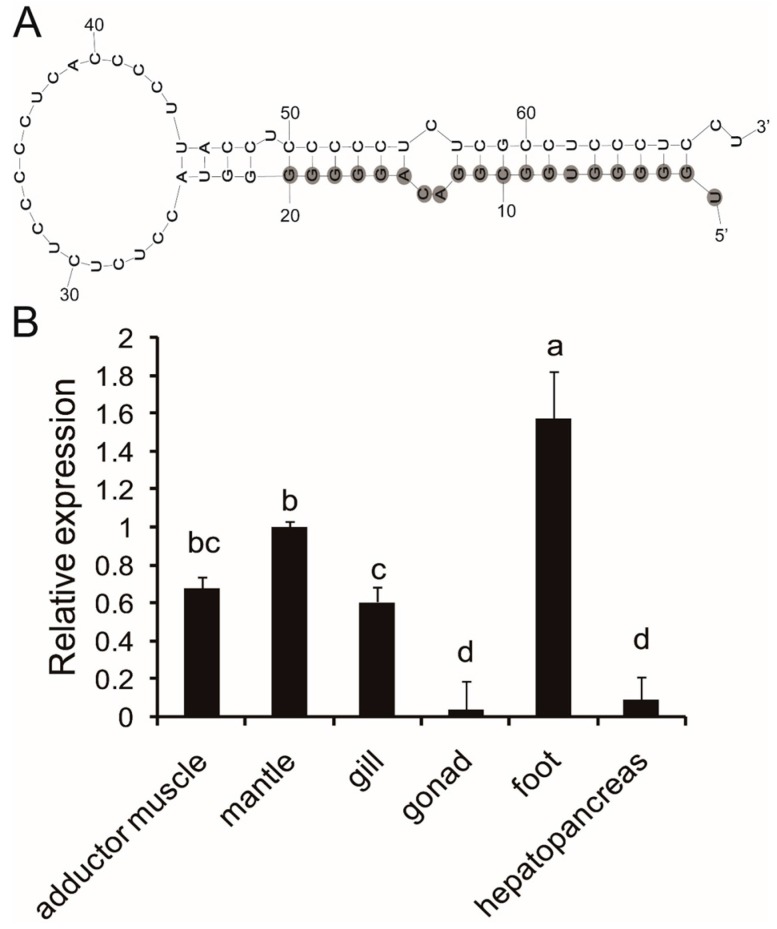
Sequence and expression analysis of pm-miR-2305. (**A**) Hairpin structure of precursor pm-miR-2305 predicted by the M-fold program; the nucleotides with gray background were the mature sequence of pm-miR-2305; (**B**) tissue expression patterns of pm-miR-2305 detected by Stem-loop qRT-PCR. The significant difference is indicated by different letters (*p* < 0.05).

With the use of the local BLASTN program, the mature sequence of pm-miR-2305 was compared against the transcriptome database of *P. martensii* [9]. Results showed that only Unigene21313 contained the mature sequence of pm-miR-2305. The sequence of Unigene21313 is presented in Figure S1. M-fold was used to predict the secondary structure of Unigene21313. As shown in Figure 1A, pm-miR-2305 had a characteristic hairpin structure. This hairpin structure was 96 bp. The mature miRNA, the same as bta-miR-2305 from Bos taurus, was at the 5′ stem of the hairpin. The miRNA:miRNA* duplex mismatches were four (Figure 1A).

### 2.2. Expression Analysis of Pm-miR-2305

Stem-loop qRT-PCR is a reliable method to detect and measure the expression levels of miRNAs to validate the presence of the identified pm-miRNA. We performed Stem-loop qRT-PCR analysis to determine the tissue-specific expression of mature pm-miR-2305. Results showed that pm-miR-2305 was constitutively expressed in all the tissues (adductor muscle, gill, mantle, hepatopancreas, foot, and gonad) of *P. martensii* and was highly expressed in the foot (Figure 1B).

### 2.3. Functional Analysis of Pm-miR-2305 in Nacre Formation

**Figure 2:**
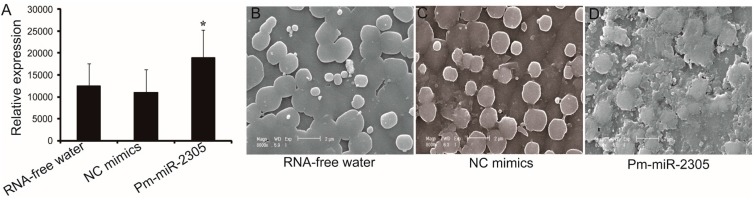
Effect of pm-miR-2305 on nacre formation. (**A**) Expression of pm-miR-2305 detected by Stem-loop qRT-PCR after injection of RNA-free water, NC mimics, and pm-miR-2305 mimics; (**B**–**D**) SEM images of the internal nacreous surface layers in the groups injected with RNA-free water (**B**); NC mimics (**C**); and pm-miR-2305 mimics (**D**) (Magnification is 8000×). (* *p* < 0.05; error bars correspond to mean ± SD).

To further investigate the function of pm-miR-2305, we over-expressed pm-miR-2305 *in vivo* by mimics injection into the muscle of *P. martensii* (two years old). Controls were groups injected with RNA-free water and NC mimics. Eight days after the first injection, Stem-loop qRT-PCR was employed to measure the expression of pm-miR-2305 in the mantle. The expression of pm-miR-2305 was up-regulated in the group injected with pm-miR-2305 mimics by approximately 1.5- and 1.7-fold compared with those in the groups injected with RNA-free water or NC mimics, respectively (Figure 2A). We observed the microstructure of nacre from each group using scanning electron microscopy (SEM). The nacre in the control groups had the same normal orderly type of microstructure (Figure 2B,C), whereas disorderly nacre growth was observed in the group injected with pm-miR-2305 mimics (Figure 2D). These results indicated that pm-miR-2305 participated in nacre formation.

### 2.4. Target Analysis of Pm-miR-2305

**Figure 3:**
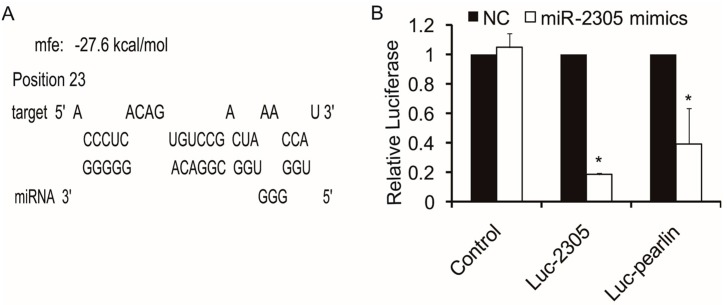
Regulatory target analysis of pm-miR-2305. (**A**) Target interaction predicted by RNAhybrid; (**B**) Pm-miR-2305 mimics significantly down-regulated the luciferase activity of the reporter containing the 3′UTR of the pearlin gene detected by dual luciferase analysis. Luc-2305: luciferase reporter plasmid containing the complementary sequence of pm-miR-2305. Luc-pearlin: luciferase reporter plasmid containing the 3′UTR of the pearlin gene. (* *p* < 0.05; error bars correspond to mean ± SD).

The next goal was to identify the potential regulatory targets of pm-miR-2305. We performed target analysis between pm-miR-2305 and the reported nacre-formation-related genes, such as nacrein, pearlin, pif-177, and dermatopontin. The lowest minimum free energy (MFE) was between pm-miR-2305 and the nacre-formation-related gene pearlin. As shown in Figure 3A and Figure S2, the MFE of the interaction between pm-miR-2305 and pearlin was −27.6 kcal/mol. These results suggested that a potential target of pm-miR-2305 was pearlin. As shown in Figure S3, the pearlin gene was detected with high expression in the mantle pallium compared with mantle edge. Complementary to pearlin expression, pm-miR-2305 was highly expressed in the mantle edge, which provided evidence that pearlin was negatively regulated by pm-miR-2305.

### 2.5. Target Verification between Pm-miR-2305 and Pearlin by Dual Luciferase Assay

To demonstrate that pearlin was negatively regulated by pm-miR-2305, we generated luciferase reporters containing the 3′UTR of the pearlin gene. We also constructed the luciferase reporters containing the complementary sequence of pm-miR-2305 to validate the activity of pm-miR-2305 in HEK-293T cells. These reporter plasmids were transfected into HEK-293T cells with pm-miR-2305 mimics or the control. After 24 h of incubation, the cells were subjected to luciferase assays. As shown in Figure 3B, pm-miR-2305 mimics significantly reduced luciferase activity to 18% in the reporter containing the complementary sequence of pm-miR-2305, indicating that pm-miR-2305 was functional in HEK-293T cells. The luciferase activity of the reporter containing the 3′UTR of the pearlin gene was also reduced to approximately 39%, suggesting that pearlin could be regulated by pm-miR-2305 (Figure 3B).

### 2.6. Pearlin Antibody Preparation

**Figure 4:**
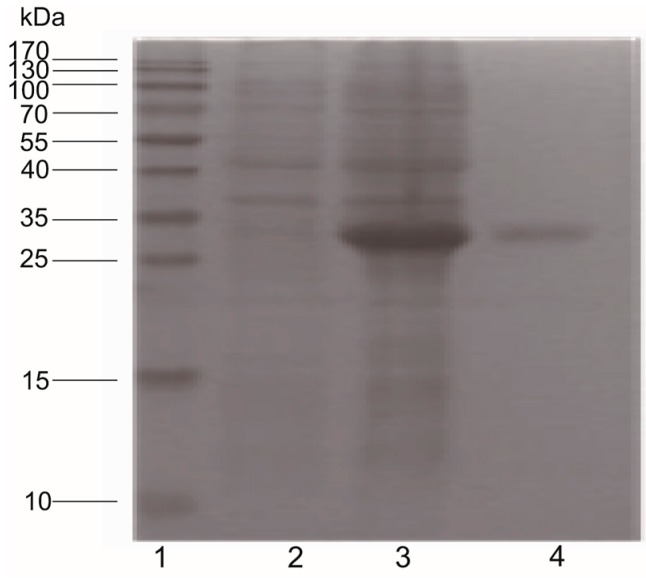
Antiserum preparation of pearlin in *E. coli* BL21. Lane 1: protein molecular marker; Lane 2: pET-pearlin without IPTG induction; Lane 3: pET-pearlin with IPTG induction; Lane 4: recombinant pearlin purified by a pre-charged HisTrap HP chelating affinity column.

To further demonstrate that pearlin was a target of pm-miR-2305, we prepared the antibody of pearlin. Sequencing revealed that the pearlin gene was correctly cloned into the expression vector pET-32a. After isopropyl-β-d-1-thiogalactopyranoside (IPTG) induction, bacteria harboring pET-pearlin produced the recombinant proteins at the size of 33 kDa (Figure 4).

### 2.7. Down-Regulation of Pearlin Protein after the Over-Expression of Pm-miR-2305

**Figure 5:**
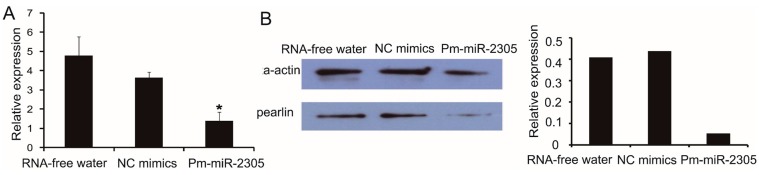
Expression of pearlin after pm-miR-2305 mimics injection. (**A**) Expression of pearlin mRNA in the mantle tissue by qRT-PCR; (**B**) Expression of pearlin protein in the mantle tissue by Western blot. Histogram shows the gray scale quantitative analysis for Western blotting using Gel-pro software (* *p* < 0.05; error bars correspond to mean ± SD).

We also analyzed the expression of pearlin mRNA by qRT-PCR using the same samples prepared in method 4.4. Notably, pearlin mRNA in the group injected with pm-miR-2305 mimics was down-regulated to approximately 29% and 39% compared with those in the groups injected with RNA-free water and NC mimics, respectively (Figure 5A). Furthermore, Western blot analysis demonstrated that the pearlin protein expression in the group injected with pm-miR-2305 mimics was inhibited by approximately 88% compared with those in the control groups (Figure 5B).

## 3. Discussion

MiRNAs are post-transcriptional regulators involved in nearly all known biological processes in distant eukaryotic clades [10]. More than a third of the protein-coding genes are predicted to be under the control of human miRNAs [11]. Recently, 35,828 mature miRNAs have been identified in 223 species (http://www.mirbase.org/, release 21). Their discovery and functional characterization have broadened the understanding of the biological regulatory mechanisms in animals and plants [12]. Pm-miR-2305, identified in *P. martensii* by deep sequencing [8], shares sequence homology with miR-2305, which was first identified in bovine [13]. Generally, a short sequence, defined as a noncoding “ideal” miRNA, should be expressed as a distinct transcript of about 22 bp and should originate from a 60- to 70-nucleotide RNA precursor with a characteristic hairpin structure that does not contain large internal bulges or loops. A mature miRNA should occupy the stem part of the hairpin [14]. Pm-miR-2305 was contained in a unigene from the transcriptome of *P. martensii* [9]. This unigene has no annotation information by BLASTX analysis, indicating that pm-miR-2305 may be a noncoding RNA. Secondary structure analysis showed that pm-miR-2305, similar to bta-miR-2305, was at the 5′ stem of the characteristic hairpin, verifying the existence of pm-miR-2305 in the genome of *P. martensii*.

MiRNA are negative regulators of gene expression by binding to specific sequences within a target mRNA. A single miRNA could have hundreds of target mRNAs [15]. Increasing evidence suggests some miRNAs, such as let-7, has universal functions and play crucial roles in cell differentiation, proliferation, growth, apoptosis and immune response as its various target mRNAs [16,17,18]. miR-125b, which was reported to participate in bone formation by targeting Smad4, was also involved in the regulation of immune response, apoptosis and cell proliferation [19,20,21,22]. The tissue expression patterns showed pm-miR-2305 was constitutively expressed in all the tissues of *P. martensii*, indicating the wide function and authenticity of pm-miR-2305 in *P. martensii*. In our previous research, pm-miR-2305 was predicted to participate in regulating nacre formation by targeting some matrix protein genes [8]; however, this prediction requires further experimental elucidation. Nacre, also called “mother of pearl,” is the inner nacreous layer of the shell and is a classical product of biomineralization [23,24]. Among the nacre components, matrix proteins secreted by the epithelial cells of the mantle are crucial in controlling crystal orientation, crystal nucleation, and mineral polymorph selection [25,26]. Inhibiting the expression of some matrix proteins can disrupt crystal polymorphisms and lead to disorderly growth of nacre [1,27]. To further explore the function of pm-miR-2305, we disrupted the expression of pm-miR-2305 by injecting pm-miR-2305 mimics into *P. martensii*. Surprisingly, disorderly growth of nacre was observed in the group injected with pm-miR-2305 mimics, indicating that pm-miR-2305 was involved in nacre formation. Generally, the function of miRNA is mediated by silencing target genes. Our previous research indicated the potential target genes of pm-miR-2305 [8]. In the present study, we focused on the nacre-formation-related genes and identified pearlin as the most potential target. Pearlin, one member of the N16 family that was isolated from a nacreous layer, is a matrix protein that is presumed to function as a template for nucleation in the initial step during nacre formation [28,29]. To further verify the interaction between pm-miR-2305 and pearlin, dual luciferase reporter gene assay was performed using HEK-293T cell lines, as molluscan cell lines are not commercially available. HEK-293T is a human cell line with high transduction efficiency. To identify the target gene of pm-miR-2305, first we had to confirm that pm-miR-2305 was active in HEK-293T. Thus, we constructed the luciferase reporter containing the complementary sequence of pm-miR-2305. Strikingly, pm-miR-2305 mimics could reduce the luciferase activity of this reporter, indicating that pm-miR-2305 was functional in HEK-293T. Meanwhile, the luciferase activity of the reporter containing the 3′UTR of the pearlin gene was also significantly reduced by pm-miR-2305 mimics, suggesting that the pearlin gene could be regulated by pm-miR-2305. Thus, we proposed that the effect of pm-miR-2305 *in vivo* may be mediated by inhibiting the expression of pearlin. To validate this hypothesis, we first detected the expression of pearlin after the over-expression of pm-miR-2305. Notably, the pearlin gene was significantly down-regulated in the group injected with pm-miR-2305 compared with that in the control. Preliminary studies of miRNA targets suggested that only the protein levels of the regulated targets decreased in animals, whereas the levels of the target mRNAs were not affected [30]. However, an increasing number of reports showed that altering miRNA expression in cells or tissues can cause significant changes in target mRNA levels, thereby suggesting that miRNAs can also induce mRNA destabilization [31,32]. To further determine the target relationship between pm-miR-2305 and pearlin, we detected the expression of the pearlin protein in *P. martensii* injected with pm-miR-2305 mimics. As expected, the expression of the pearlin protein in *P. martensii* injected with pm-miR-2305 mimics was also notably inhibited compared with that in the control, confirming our assumption that pm-miR-2305 participated in nacre formation by targeting pearlin.

Finally, based on the transcriptome database of *P. martensii*, we obtained the precursor sequence of pm-miR-2305 and analyzed its tissue expression profile. The ectopic expression of pm-miR-2305 *in vivo* disrupted the normal growth of nacre. Both dual luciferase reporter gene assay and Western blot analysis indicated that the function of pm-miR-2305 in nacre formation may be mediated by targeting pearlin. Our study is the first to provide evidence that miRNA participates in nacre formation in *P. martensii*. To better understand the regulatory network in nacre formation, further studies on the detailed regulatory mechanism of pm-miR-2305 are required.

## 4. Experimental Section

### 4.1. Experimental Samples and RNA Extraction

Pearl oysters *P. martensii* (about 2 years old) were collected from Liushagang, Zhanjiang, Guangdong Province of China. The tissues of the hepatopancreas, adductor muscle, mantle, gill, foot, and gonad of *P. martensii* were collected and stored in liquid nitrogen until used. Total RNAs were extracted using Trizol reagent (Invitrogen, Carlsbad, CA, USA). The quantity of RNA was determined by measuring OD260/OD280 with a NanoDrop ND1000 Spectrophotometer. The integrity of RNA was determined by fractionation on 1.2% formaldehyde-denatured agarose gels.

### 4.2. Precursor Prediction of Pm-miR-2305

The search procedure for a potential precursor of pm-miR-2305 was similar to a previously described method used for the identification of potential new miRNAs [33]. First, local BLASTN was performed to compare the mature sequence of pm-miR-2305 against the transcriptome database of *P. martensii* [9]. The obtained unigenes containing the mature pm-miR-2305 were annotated by BLASTX analysis. The secondary structure of the unigene sequence was predicted by M-fold program (http://mfold.rna.albany.edu/).

### 4.3. Expression Analysis of Pm-miR-2305 and Pearlin

Stem-loop qRT-PCR analysis was employed to determine the tissue-specific expression of pm-miR-2305 with U6 as an internal control. Stem-loop RT primers and real-time PCR primers were designed as previously described by Chen *et al.* [34]. The primers used in this analysis are listed in Tables S1 and S2.

### 4.4. Over-Expression of Pm-miR-2305 in Vivo

Pm-miR-2305 (UGGGGGUGGCGGACAGGGGG, GenePharma, Shanghai, China) and NC mimics (UCACAACCUCCUAGAAAGAGUAGA, GenePharma, Shanghai, China) were diluted to 0.1 μg/μL using RNase-free water. Exactly 100 μL of RNA-free water, miR-2305, and NC mimics solution were separately injected into the muscle of *P. martensii* for the first time, and the same doses were injected 4 days after the first injection. Ten individuals were used in each group. The mantle was acquired 8 days after the first injection and immediately stored in liquid nitrogen until usage. The shells were cut into pieces, coated with carbon and observed by FEIQuanta 200 scanning electron microscope (SEM).

### 4.5. Construction of Luciferase Reporter Plasmids

Pearlin sequence was downloaded from the NCBI database. The 3′UTR of pearlin gene was amplified using PCR and cloned into the pMIR-REPORT vector between the Spe I and Hind III sites, immediately downstream from the Renilla luciferase gene. The primers used for PCR were as follows: forward primer CTAGACTAGTCTTCCTTGATTCTTGAATTTTAC containing one Spe I site and reverse primer CCCAAGCTTAATCTGGCTTCATACTTTACTATAT containing one Hind III site. The sequence for luciferase reporters containing the complementary sequence of pm-miR-2305 was as follows: forward CTAGTCCCCCTGTCCGCCACCCCCAtactaCCCCCTGTCCGCCACCCCCAtactaCCCCCTGTCCGCCACCCCCAa containing one Spe I site and reverse AGCTTTGGGGGTGGCGGACAGGGGGtagtaTGGGGGTGGCGGACAGGGGGtagtaTGGGGGTGGCGGACAGGGGGa containing one Hind III site.

### 4.6. Target Analysis between Pm-miRNAs and Nacre Formation-Related Genes

Nacre formation-related genes were downloaded from NCBI GenBank. The target analysis between miRNA and the 3′UTR of the nacre formation-related genes was performed using RNAhybrid [35]. RNAhybrid can be used to determine the most favorable hybridization site between miRNA and mRNA. This tool can also be used to calculate the free energy in the hybridization site. A more negative free energy indicates a more stable duplex. Only less free energy (<−15 kcal/mol) was considered to have a potential interaction between miRNA and mRNA in the current study.

### 4.7. Luciferase Assay

Luciferase reporters (pMIR-REPORT-miR-2305, pMIR-REPORT-3′UTR/pearlin) and pm-miR-2305 or NC mimics were co-transfected into HEK-293T cells using Lipofectamine™ 2000 (Invitrogen). At 24 h after the transfection, luciferase activity was measured using a dual luciferase assay kit (Promega, Madison, WI, USA) according to the manufacturer’s protocol.

### 4.8. Protein Expression, Purification, and Antiserum Preparation

The forward primer used for expression was CGGGATCCGCTTTCCGTACGAAGTGC containing one BamH I site, and the reverse primer was CCCAAGCTTTTACTTGTCATACCGTTCATCG containing one Hind III site. The PCR products were digested with BamH I/Hind III and then inserted into a prokaryotic expression vector pET-32a (Novagen, Madison, WI, USA). The prokaryotic expression vector pET-32a/pearlin was transformed into *Escherichia coli* BL21. Protein expression was induced with 1 mM IPTG at 37 °C. After 4 h of induction, bacterial cells were harvested. The recombinant fusion protein was purified by a pre-charged HisTrap HP chelating affinity column (Amersham, Piscataway, NJ, USA) according to the manufacturer’s instruction. Antiserum was produced in rabbits. A New Zealand white rabbit was immunized with 2 mg of purified proteins that were homogenized in complete Freund’s adjuvant (Sigma, St. Louis, MO, USA). The booster injection in incomplete Freund’s adjuvant (Sigma) was administered after a week. Rabbit blood was collected by carotid puncture after the fourth immunization. An antiserum was prepared according to the method described by Cai *et al.* [36].

### 4.9. Western Blot

Western blot analysis was performed by using the prepared antiserum as the primary antibody (1:500), as previously described [37]. β-Actin was used to confirm equal loading of proteins. Human β-actin antibody was supplied by PeroTech (Scarborough, ON, Canada).

### 4.10. Statistical Analysis

The data were analyzed using one-way ANOVA in SPSS 19.0 (IBM, Chicago, IL, USA). A *p*-value less than 0.05 (*p* < 0.05) was considered as statistically significant.

## Supplementary Materials

Supplementary materials can be found at http://www.mdpi.com/1422-0067/16/09/21442/s1.
