# Supplementary material for: MicroRNA, Pm-miR-2305, Participates in Nacre Formation by Targeting Pearlin in Pearl Oyster Pinctada martensii
Source: Int J Mol Sci. 2015 Sep 7;16(9):21442–53. doi: 10.3390/ijms160921442 (PMC4613262; doi:10.3390/ijms160921442)
Supplement: Supplementary file 1 [file ijms-16-21442-s001.pdf]

Supplementary Information

Table 1. Primers Sequences used in stem-loop qRT-PCR.

Name	RT Primer	qPCR-Antisense Primer	qPCR-Sense Primer
miR-2305	GTCGTATCCAGTGCGTGTCGTGGAGTCGGC AATTGCACTGGATACGACCCCCCTGT	GGGGGTGGCGGACAGGGG	GTGCGTGTCGTGGAGTC
U6	GTCGTATCCAGTGCGTGTCGTGGAGTCGGC AATTGCACTGGATACGACAAAAATATGG	TGCGTGTCGTGGAGTC	ATTGGAACGATACAGAG AAGATTAG

Table 2. Primers Sequences used in qRT-PCR.

Primer Name	Primer Sequence (From 5′ to 3′)	Application
Pearlin-F	TCATACTGCTGGATACCCTA	qRT-PCR
Pearlin-R	GTCCACATCTTAGCCACTCA	qRT-PCR
β-actin-F	GTGTAAGGCGGGGTTTGCT	qRT-PCR
β-actin-R	GGGTCCTTCAGCGTTAGTATCTT	qRT-PCR

ATGATATTGATTAACTTGTTCATGTCACAGTATGTTCCGTGTTATGCTCATAGAGCAACCGCATATCATCATTTTA
GTGTATTTTAGTCTTGAGTTTAAGTTTAATTTTGTAGGCTTAGCCATAGTACATATGGGGGTGGCGGACAGGG
GGGGTACCTCTCTCCCCCTCACCCCTTACCTCCCCCTCTCGCCTCCCTCCTCCCGCCCCTCTTCCATCCCCTCCC
TCCCATGTTTCGCCCTTCCCTCCCTCCTCCCTTTCTCCCTCCTCTCTTTCTCCCTCCTCTCGCCCCCTTCCTTCTG
CCTCCCTTTCACCCTGTCCACCCCTCACATATAGTAGATGAACGAACGGTTGGACTAATATGGTTCCCTGTGCT
TTAAGCGCAAAGTGTATGTATTGATGTTTGATGTGAGTTCTACTAATTTTTTGTGTGTTGTTGTTTTATCGTGCT
ATAAGCGCATTGTTAAAGCGAGTTAAAACCGCAAAAAAACTTGTAATTATAAAAGACACATAAATACCATTTA
TGATTATGTAAATAATTCAATAATTCGCCG

Figure S1. Sequence of Unigene21313. The nucleotides indicated in red were the mature sequence of pm-miR-2305.

a
```
target: DPT (Dermatopontin)
miRNA : miR-2305
mfe: -17.6 kcal/mol

position  99
target 5'      G    AGA       U 3'
                GUCC   UACUUCU
                CAGG   GUGGGGG
miRNA  3' GGGGGA    CG        U 5'
```

b
```
target: Pif177
miRNA : miR-2305
mfe: -24.1 kcal/mol

position  13
target 5' A    AUA     CAAA UG   A  3'
           CCCC   UGUCU     C  CUU
           GGGG   ACAGG     G  GGG
miRNA  3'      G       CG   UG   GU 5'
```

c
```
target: nacrein
miRNA : miR-2305
mfe: -26.1 kcal/mol

position  129
target 5' A  AG   G     A     U 3'
           CU   GU CGCUA CCCCU
           GG   CA GCGGU GGGGG
miRNA  3' G  GGA  G           U 5'
```

d
```
target: TIMP (Tissue inhibitor of metalloproteinases)
miRNA : miR-2305
mfe: -22.3 kcal/mol

position  11
target 5' A   ACAUCUAU    UU   UACAGAAUAU      G 3'
           CCU        UUGU  GCC          GCUCUU
           GGG        GACA  CGG          UGGGGG
miRNA  3' G               GG                   U 5'
```

e
```
target: BMP-7 (Bone Morphogenetic Protein 7)
miRNA : miR-2305
mfe: -27.0 kcal/mol

position  667
target 5' A    G        G    GU   U 3'
           UCUU UGUCCGUC AUCU  CCG
           GGGG ACAGGCGG UGGG  GGU
miRNA  3'      G                   5'
```

f
```
target : N14#1
miRNA  : mir-2305
mfe: -23.5 kcal/mol

position  2
target 5' A      CUA  GAUUCUUGAAUU         U 3'
           UUCCUG   CG            UUACCCUCA
           GGGGAC   GC            GGUGGGGGU
miRNA  3' G      AG                          5'
```

Figure S2. Target interaction between nacre formation-related genes and pm-miR-2305 predicted by RNAhybrid. Nacre formation-related genes included DPT (**a**); Pif177 (**b**); nacrein (**c**); TIMP (**d**); BMP-7 (**e**) and N14#1 (**f**).

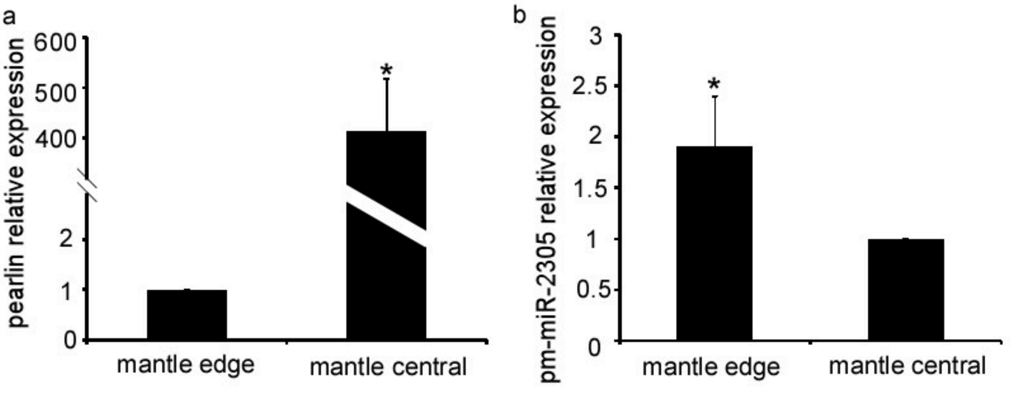

Figure S3. Expression of pearlin (**a**) and pm-miR-2305 (**b**) in the mantle edge and mantle central. (* $p < 0.05$; error bars correspond to mean ± SD).
